# Influence of cortical bone and implant design in the primary stability of dental implants measured by two different devices of resonance frequency analysis: An *in vitro* study

**DOI:** 10.4317/jced.56014

**Published:** 2020-03-01

**Authors:** David Chávarri-Prado, Aritza Brizuela-Velasco, Markel Diéguez-Pereira, Esteban Pérez-Pevida, Antonio Jiménez-Garrudo, Iratxe Viteri-Agustín, Alejandro Estrada-Martínez, Oier Montalbán-Vadillo

**Affiliations:** 1 Department of Surgery and Medical-Surgical Specialties, Faculty of Medicine and Dentistry, University of Oviedo, Oviedo, Spain; 2 Department of Surgery, Faculty of Medicine, University of Salamanca, Salamanca, Spain; 3 Department of Surgery, Gynecology and Obstetrics. Faculty of Sport and Health Sciences, University of Zaragoza, Huesca, Spain

## Abstract

**Background:**

This study aimed to evaluate the effect of the implant design and the presence of cortical bone in the primary stability, as well as analyze the differences between the stability measurements obtained by two different resonance frequency analysis (RFA) devices.

**Material and Methods:**

A total of 80 Klockner implants of two different models [40 Essential Cone implants (group A) and 40 Vega implants (group B)] were used. The implants were placed in two polyurethane blocks that simulated the mechanical properties of the maxillary bone. One block featured a layer of cortical bone that was absent from the other block. The primary stability of all implants was measured by insertion torque and RFA using two different devices: Penguin RFA and Osstell IDX.

**Results:**

Primary stability was superior in the cortical bone in both torque and RFA. In the block containing cortical bone, group A implants obtained a greater insertion torque than did group B. The insertion torque was lesser in the bone lacking cortex. Regarding the ISQ of the implants, group A presented higher values in the block with cortical bone, but the values were lower in the block without cortical bone. There were no significant differences between the values obtained from the Osstell IDX and Penguin RFA.

**Conclusions:**

The presence of cortical bone positively influences the primary stability of dental implants. The design of the implant also has a statistically significant influence on implant primary stability, although the impact depends on whether there is coronal cerclage or not. There were no statistically significant differences in the implant stability measurements obtained by two different devices.

** Key words:**Implant stability, resonance frequency analysis, torque, osstell, penguin, cortical.

## Introduction

Recently, implant stability has been defined as the absence of clinical mobility under a given load, and it is considered one of the main requirements for the achievement and maintenance of the osseointegration of a dental implant (1,2). This stability can be influenced by multiple factors, such as the density of the recipient bone, the surgical technique or the design of the implant itself (3). Regarding bone density, several authors have shown that the presence of cortical bone increases the primary stability of the implant, since it provides one or several anchorages in a bone of a much greater density compared to implants fixed only in the trabecular bone, which is much less dense (4-10). However, it would be appropriate to discuss which of the two cases would produce the best osseointegration or the best biomechanical behaviour in response to the load. This question is derived from the better mechanical properties offered by the cortical bone due to its higher mineral content and higher density, but conversely, it shows much poorer vascularity and significantly lower cellularity, which could influence its secondary stability (11).

 Secondary, or biological, stability is the result of the physiological processes of bone apposition and remodelling on the surface of the implant, which leads to osseointegration (12-15). It is a dynamic parameter that can vary throughout the functional life of the implant, unlike primary, or mechanical, stability, which is a static parameter obtained in a single moment of time on the day of its placement (3,16,17). Primary stability is a purely mechanical concept that results from the friction or resistance between the bone and the implant upon insertion (18,19).

Over the years, different methods have been used for the measurement of stability (15). These methods can be classified as invasive and non-invasive. Invasive methods include histology, which evaluates the bone-to-implant contact percentage, and disinsertion torque (20). Both methods require removal of the implant; therefore, they present no clinical applicability. For clinical applications, non-invasive methods must be used, such as percussion testing, which is unreliable and highly subjective, since it depends on interpretation by each clinician of the frequency of the sound emitted by the implant when it is hit with the handle of a mirror (21). Therefore, Brizuela *et al.* (22) proposed a method for the measurement of implant stability based on an objective interpretation of this frequency, but to date, this method has not yet been developed for use in clinical practice. A different non-invasive method for measuring implant stability is the periotest. The periotest is an objective method developed by Schuttle in 1993, created to measure the mobility of teeth in periodontal disease. The method uses an electronic device with a punch that pushes the tooth and measures its lateral displacement, delivering objective stability values on a scale from -8 to +50 (23-25). Inconveniently, as this method was developed to measure tooth mobility, it presents insufficient sensitivity to measure micromotion in implants, since teeth, being surrounded by the periodontal ligament, present significantly greater mobility than do implants (26-28).

Another non-invasive method is measurement of the insertion torque, which is widely referenced in the literature and currently very widespread clinically (13,29). The torque is the resistance required to move the implant in the bone forward in the apical direction. Although it has been shown to maintain an inverse relationship with micromotion, statistically it is not linearly but exponentially maintained. Thus, at low torque values, small increases in torque generate large reductions in micromotion, while for high torque values, large increases in torque barely reduce the degree of micromotion (18). Insertion torque can only be measured once, at the time of implant placement. Since it does not allow subsequent remeasurement of the stability, it does not facilitate controlling and monitoring the biological stability of the implant.

**Figure 1:**
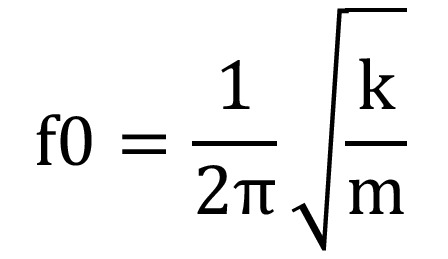
Formula.

 Thus, resonance frequency analysis (RFA) is currently the only non-invasive method for objectively measuring and monitoring implant stability (17). The method is based on detection of the natural frequency of vibration of the implant within the bone, which depends on the rigidity of implant attachment and its mass, as can be deduced from the following formula, where “f0” is the natural frequency of vibration, “k” is the stiffness constant, and “m” is the mass, (Fig. 1).

Therefore, the RFA device relies on the physical phenomenon of resonance to determine the natural frequency of vibration of each implant and thus provide the clinician an implant stability quotient value (ISQ) on a scale of 0 to 100 (30).

Currently, there are several devices on the market that use RFA as a method of measuring implant stability, such as the Osstell IDX (Osstell, Gothembourg, Sweden) and the Penguin RFA (Integration Diagnostics Sweden AB, Gothenburg, Sweden) (31,32).

The objective of the present *in vitro* experimental study was to determine the influence of the presence of cortical bone and how the design of the implant impacts implant stability, as well as to compare the results of the measurements obtained using two different RFA devices.

## Material and Methods

-Implant design 

**Figure 2:**
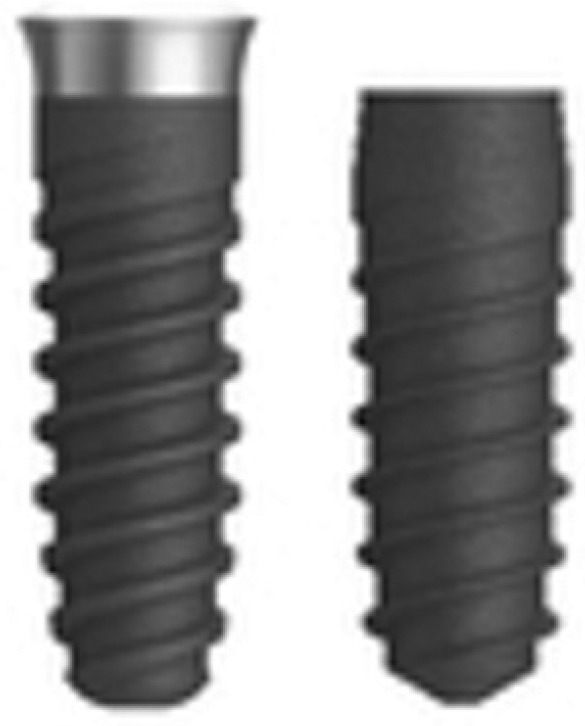
Shows the two types of implants used in the study: Klockner Essential Cone and Klockner Vega.

Eighty Klockner internal connection implants were used (Klockner Implant System, Madrid, Spain), all with the same diameter and length (4×10 mm), divided equally into two model implant models with very different designs. Forty of the implants were used in the Essential Cone® model (Group A), while the remaining 40 were placed in the Vega® model (Group B). The main difference between these two implant models is that the Vega implant is a “bone level” implant model with a surface treatment across the entire model, while the Essential Cone implant is of the “tissue level” type and features a neck of 1.5 mm of fully polished, machined surface, which must be maintained in a suprabony position (Fig. 2).

-Bone specimens 

**Figure 3:**
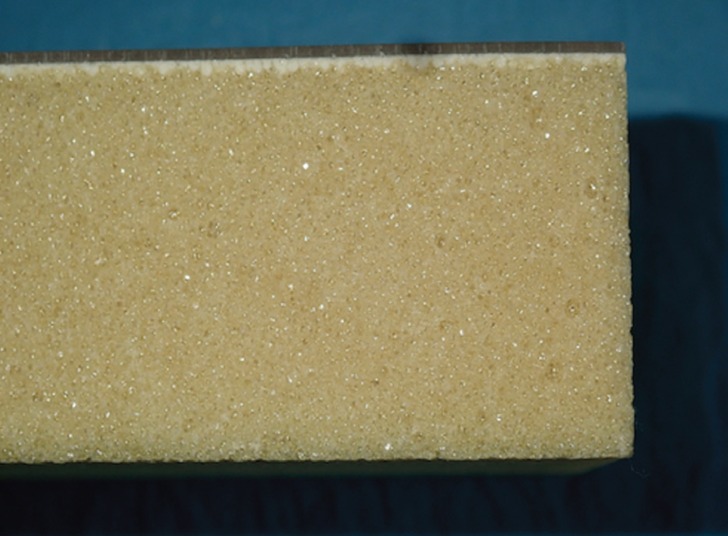
Shows one of the polyurethane blocks used in the study, where the imitation of cortical and trabecular bone can be observed.

To simulate the supporting bone, two polyurethane blocks (Sawbones®, Pacific research Laboratories Inc., Washington, USA) were used, with a density of 0.32 gr/cm3, which reproduces the mechanical properties of the human posterior maxilla (33,34). One of the blocks had a 1-mm layer of a resin with a much higher density (1.64 gr/cm3) on the top of its surface, simulating the presence of cortical bone, while the other block consisted solely of trabecular bone (Fig. 3).

-Drilling and implant insertion protocol 

For implant placement, the milling protocol recommended by the manufacturer (Klockner Implant System, Madrid, Spain) was followed. Despite two different implant models being used, the same milling sequence was suggested for both. This sequence involved passing a lanceolate bit to mark the bed, followed by a pilot cut of 1.8 mm in diameter and another 2.8 mm cut for expansion. From this point, the specific milling for the 4-mm-diameter implants was based on passing a profile bit and finishing the process with 3.6-mm-diameter mill work. For this study, all 80 implant beds were prepared by the same researcher performing the complete sequence.

-Stability measurements 

For measurement of the implant stability, two different methods were used. First, the insertion torque of each implant was measured with a correctly calibrated digital torque meter AMG 100N (Mecmesin, Barcelona, Spain). Then, the stability of the implants were measured using two RFA devices of different brands: Osstell IDX® and Penguin RFA®. To compare the similarity of the measurements from the two devices, 4 measurements were conducted per implant. The add-on devices presented by each brand were also used: Osstell + Smartpeg, Osstell + Multipeg, Penguin + Multipeg and Penguin + Smartpeg.

**Figure 4:**
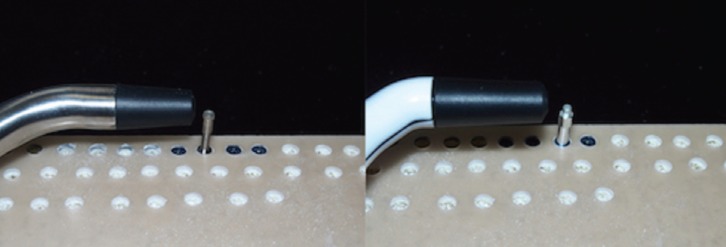
Shows the procedure of stability measurement with RFA using an Osstell IDX with Multipeg device (left) and using a Penguin RFA device with Smartpeg (right).

 Each measurement was conducted in the same orientation, and both the Multipeg and the Smartpeg were tightened by applying the force recommended by the manufacturer (6-8 N) using a torque wrench with an ad hoc screwdriver (Fig. 4). Since the Multipeg features titanium fabrication and can be used repeatedly as many times as desired, it was used for the entire study. However, the Smartpeg is made of aluminium and has limited use. Since the material of the device is softer than that of the implant, the thread deteriorates and the measurement of the ISQ loses reliability. The manufacturer recommends a maximum of 10 uses per Smartpeg, but a study published by Brizuela *et al.* in 2015 guaranteed at least 20 uses; thus, for this work, 4 Smartpegs were used, one for each study group (35).

-Statistical analysis 

Statistical analysis of the data was carried out by applying descriptive and inferential statistic techniques. For description of the continuous variables, means and standard deviations were used. Normality was checked using the Shapiro-Wilk test. Given the lack of normality, comparison between categories was conducted through the Kruskal-Wallis test, considering the equality of medians between groups as a null hypothesis. Finally, the strength of the relationships between variables was studied using the Pearson correlation coefficient.

## Results

The results of this *in vitro* experimental study revealed that the primary stability of each implant was significantly higher in the block that simulated the presence of cortical bone than in that with only trabecular bone. The insertion torque of the implants of group A was 33.52 ± 6.29 N•cm in the cortical bone and 12.38 ± 2.20 N•cm without cortical bone, while in group B, the torque was 20.47 ± 3.74 N•cm with cortical bone and 14.01 ± 2.47 N•cm without cortical bone. Through analysis with a two-way ANOVA test, it was observed that both the type of implant (*p* < 0.001) and the presence of cortical bone (*p* < 0.001), as well as the interaction of both variables, were significant (*p* < 0.001).

Regarding the design of the implant, the torque results showed statistically significant differences between both groups when they were placed in the block with cortical bone (33.53 ± 6.29 N•cm vs 20.47 ± 3.74 N•cm) (*p* < 0.001) and when they were placed in bone without cortical cerclage (12.38 ± 2.20 N•cm vs 14.01 ± 2.47 N•cm) (*p* = 0.034).

Table 1 shows the insertion torque of group A implants with and without cortical bone, while Table 2 shows the same information for group B.

Regarding torque and RFA note that there was a strong correlation between the ISQ values obtained using the different devices (Osstell or Penguin) and the different add-ons (Multipeg or Smartpeg), with all of these correlations being above 0.95, while the correlation values of the torque with the ISQ variables ranged between 0.81 and 0.87 (Table 3).

**Table 1:**
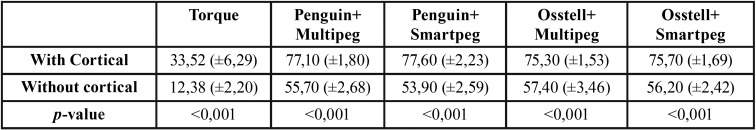
Group A (EC). Mean values and standard deviation, as well as comparison between groups, with and without cortical bone.

**Table 2:**
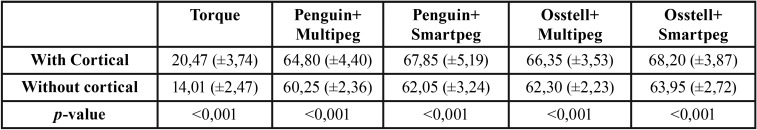
Group B (VEGA). Mean values and standard deviation, as well as comparison between groups, with and without cortical bone.

**Table 3:**
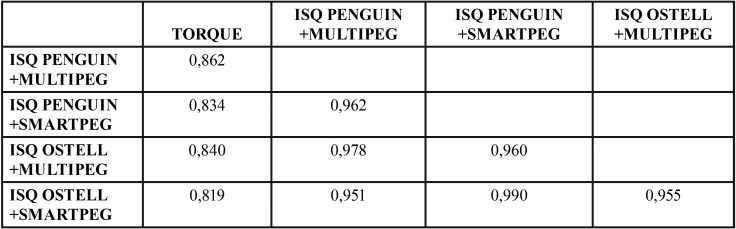
Correlation coefficients relating the variables TORQUE and ISQ with different devices and different abutments.

In addition, a strong influence of the presence or absence of cortical bone on the ISQ values was observed in the implants of both group A and group B ( Table 1, Table 2), with statistically significant differences in each torque and ISQ-analysed variable. For example, considering the measurements obtained using Penguin with Multipeg, note that the ISQ in the cortical block was 77.07 ± 1.80 for the implants of group A and 64.80 ± 4.40 for the implants of group B, while in the block without cortical bone, ISQ values of 55.70 ± 2.68 for the implants of group A and 60.25 ± 2.36 for those of group B were presented.

The average torque value of the implants of group A was 22.90 ± 11.70, while that of group B was 17.24 ± 4.53, indicating statistically significant differences between the groups (*p* < 0.006). In the case of ISQ, the results obtained were compared with those from the two devices and add-ons, without indication of statistically significant differences between the groups (*p* = 0.696).

## Discussion

To carry out this study, polyurethane blocks were used to reliably reproduce the mechanical properties of the human maxillary bone. Previous studies have demonstrated that artificial bone blocks were useful to simulate cortical and trabecular bone conditions in a controlled manner. Multiple published articles have used this type of study model (4,33,36-40). The advantage of this type of block over the animal bone ex vivo model is the homogeneity of the samples in terms of macroscopic characteristics; therefore, using this type of *in vitro* model guarantees that each implant is placed in an identical bone model with the same density. The density and thickness of the artificial bone material was chosen with consideration of the human trabecular bone and combined cortical bone.

This study demonstrates the influence of cortical anchorage on the primary stability of dental implants, expressed both in insertion torque and in ISQ values, and the results agree with those of several previously published works. For example, in 2014, Kim *et al.* (4) placed dental implants in polyurethane blocks, as performed in this study. Likewise, they also measured implant stability by both insertion torque and RFA. The results showed that an increase in the thickness of the cortical bone portion generated an increase of both stability parameters. These findings also agree with those obtained by Miyamoto *et al.* in 2005 (5) in a clinical study wherein the existence of a strong statistical correlation was demonstrated between the cortical bone thickness and the ISQ values obtained by RFA.

The results of this study also demonstrate significant influence of the implant design on the primary stability of the implant. Upon using implants of the same diameter and length placed following exactly the same milling protocol, Essential Cone implants (Group A) obtained greater primary stability in the block with cortical bone in terms of both insertion torque (32, 93 ± 6.29 vs. 20.19 ± 3.74) and ISQ values (77.07 ± 1.80 vs. 64.65 ± 4.39 using Penguin RFA + Multipeg). This difference can be explained by the design of the cervical region of the implant. With a wider coronal zone, implants can provide greater resistance to advancement in an apical direction, requiring significantly higher torque values. From a clinical approach, obtaining very high torque values (> 40 N/cm2) is not beneficial, since as demonstrated by Brizuela *et al.* (18), these levels of torque reduce micromotion, and therefore, the odds of success of the osseointegration process are not increased. Excessive torque can also cause strong compression of the coronal cortical bone, which would result in the appearance of microfractures in and subsequent resorption of the bony trabeculae around the implant (41). In this study, the torque obtained in group A implants with cortical bone was high but did not reach a sufficient level to be considered excessive.

However, when the primary stability data obtained in the block without cortical bone was analysed, no statistically significant differences were observed in the insertion torque achieved (12.38 ± 2.20 vs. 14.01 ± 2.47) due to the greater elasticity of the trabecular bone compared to the cortical bone; therefore, although the cervical region of group A implants is wider and causes greater compression, this did not translate into an increase in stability. Interestingly, there were statistically significant differences in the primary stability measured by RFA, but in this case, the results were in favour of group B implants (55.70 ± 2.68 vs. 60.25 ± 2.36). The macrodesign of implant type B and the distance between turns favouring primary stability explain this finding. Therefore, in a clinical situation of maximum demand, such as in the absence of cortical bone, group B implants show a better response. However, in the presence of cortical bone, which presents a greater Young’s modulus and, therefore, less deformation capacity, implant type A, which has a wider neck, causes greater compression in the cervical region, thus achieving greater stability.

From a clinical approach, this finding is very significant, since it shows that an implant design like that of group B (Vega) provides high levels of primary stability as measured by RFA, which reduces the micromovement of the implant and increases the odds of success of the osseointegration process. This objective is also reached without increasing the insertion torque, which as explained above, should optimally be maintained at a moderate level.

In the comparison of the stability measurements obtained using two different RFA devices, no statistically significant differences between Penguin RFA and Osstell IDX were observed in this study, even when the add-ons screwed into the implant (Multipeg and Smartpeg) were interchanged with each other for the measurements.

 This information is very useful for the clinician, since it demonstrates that both devices can be used indistinctly, even including use of both add-ons, since the small variations presented in the results of their measurements were not statistically or clinically significant.

Recently, a clinical study was published by Becker *et al.* in which Osstell and Penguin devices were compared (32). The results of this study were in line with ours, showing that both devices are perfectly valid for the measurement of implant stability, both primary and secondary, with no significant differences between the ISQ values obtained for each implant.

## Conclusions

Based on the results obtained and despite the limitations of using an experimental *in vitro* study, the following conclusions were obtained.

1. The presence of cortical bone in the coronal zone significantly influences the primary stability of dental implants, as expressed in both insertion torque and ISQ values.

2. The design of the implant also has a statistically significant influence on implant primary stability, in terms of both insertion torque and ISQ values, although the impact depends on whether there is coronal cerclage or not.

3. A tissue-level implant design with widening in the cervical region of the implant can significantly increase the primary stability when placed in bone with cortical cerclage compared to that achieved using a bone-level implant design with a narrower cervical region.

4. The Vega implant obtains values of stability significantly superior to those of Essential Cone implants when both are placed in bone without cortex.

5. The use of two different RFA systems, such as Osstell and Penguin, causes small variations in the ISQ values but without statistical significance, demonstrating that it is not clinically relevant to suggest the use of one system over another.
